# Applying lead (Pb) isotopes to explore mobility in humans and animals

**DOI:** 10.1371/journal.pone.0274831

**Published:** 2022-10-26

**Authors:** Jane A. Evans, Vanessa Pashley, Katy Mee, Doris Wagner, Mike Parker Pearson, Delphine Fremondeau, Umberto Albarella, Richard Madgwick

**Affiliations:** 1 National Environmental Isotope Facility, British Geological Survey, Keyworth, United Kingdom; 2 British Geological Survey, Keyworth, United Kingdom; 3 Institute of Archaeology, University College London, London, United Kingdom; 4 Department of Archaeology, University of Reading, Reading, United Kingdom; 5 Department of Archaeology, University of Sheffield, Sheffield, United Kingdom; 6 School of History, Archaeology and Religion, Cardiff University, Cardiff, United Kingdom; University at Buffalo - The State University of New York, UNITED STATES

## Abstract

Lead (Pb) isotopes provide a complementary method to other provenance tools for tracking the origin and movement of humans and animals. The method is founded in the geographic distribution of Pb isotope ratios. However, unlike the Sr isotope method that is closely linked to the lithology of underlying rocks, Pb more closely reflects the tectonic regimes. This makes it particularly pertinent to use in Britain as there is major tectonic boundary (the Iapetus Suture) that runs between Berwick-upon-Tweed and the Solway Firth providing a compositional boundary in Pb isotope domains that approximates to the geographic areas of Scotland versus England and Wales. Modern pollution makes it difficult to use modern floral or faunal samples to characterize biosphere variation, and so we use geological datasets to define isoscape variation and present the first Pb isotope map of Britain. We have validated the use of these data form biosphere studies using well provenanced samples. Reference fields of diagnostic compositions, are created in μ-*T* space and these have been used in a test case to assess the geographic origins of Neolithic animals in Great Britain.

## Introduction

### Aims

Pb isotope analysis of tooth enamel is becoming an increasingly important method of analysis for constraining geographic origins in humans and animals [1–6] as it provides another dimension in the assessment of origins of populations in Britain by combining multiple isotope proxies. The primary aims of this paper are to 1) demonstrate the potential of lead (Pb) isotope analysis to provide a geographic discriminant between the two major tectonic zones of Great Britain, 2) to show that geological Pb signatures are transferred to the fauna exploiting these in the past and 3) to use the method to refine the geographic constraint of the origins of pigs found at several Neolithic henges in southern England.

## Background

### Reasons for using Pb isotope analysis

Sr isotopes have made a significant contribution to the understanding of human and animal movement and migration [7]. However, one problem with the Sr isotope system is that it cannot distinguish between the long-term build-up of ^87^Sr through time in old rocks, as opposed to the rapid build-up of ^87^Sr in younger, but rubidium-rich, rocks. Both cases result in the transmission of high ^87^Sr/^86^Sr ratios into the biosphere. Therefore it is not possible to discriminate between old terrains, such as the Baltic Shield in Scandinavia, and younger Rb-rich granite terrains such as those found in central Scotland, Iberia and central France, using Sr alone [8].

Pb is present in magmatic rocks, predominantly in the silicate mineral feldspar. It is found in moderate concentrations within the Earth’s crust and is recycled into metamorphic and sedimentary rocks via tectonic and erosional processes. Average Pb concentrations in igneous, sedimentary and metamorphic rocks range between 6 and 23 ppm [9]. Pb is mobile in the crustal environment and, for example, can be leached out of parent rocks and concentrated into Pb-rich sulphide ore deposits during metamorphic and hydrothermal geological events [10]. Such processes can separate, to an almost complete degree, daughter-product Pb isotopes from their radioactive parent U and Th isotopes and hence lock in the isotope signatures of the Pb from when it was mobilized and redeposited [10]. Ore deposits hence provide age-defined isotope composition for the source of Pb scavenged from the source rocks to produce the ore deposit. Such ore deposits are utilized from the Bronze Age and the recycling of the extracted Pb by human activity leads to anthropogenic Pb pollution and distribution and cultural focussing of the signal [11], blurring or obliterating a primary local Pb signature. The Neolithic material that is the focus of this study, however, predates human ore mining. Therefore, the uptake of Pb by pigs will be dominated by ingestion of locally sourced food and through the accidental ingestion of soils while rooting and grubbing. To this end, it is important to understand the isotope composition of locally derived labile Pb in soils of both silicate and carbonate composition, and their overlying biosphere. However, we cannot take direct measurements of Pb in the modern biosphere in a manner analogous to sampling Sr [12]. This is because of the ubiquitous blanket of modern pollutant Pb. The Pb used in petrol, as an anti-knock reagent, in the UK is imported Australia Pb which has a distinctive and very different isotope composition from geogenic [13] UK Pb and ore deposits.

Alternative methods of defining geospatial distribution of Pb isotope compositions therefore have to be found. In an American-based study, prehistoric wild animal data are suggested as the best proxy for biosphere-assimilated Pb [6] and there is much sense in this recommendation. However, such material is hard to come by in Britain. We have therefore opted to use geological data [14–20], and data from this study, and to validate this, where possible, against faunal data from a similar geographic location. This approach is open to further refinement and validation, but nevertheless constitutes a useful first step. We have used published isotope data from soil-forming rocks and minerals, in addition to galena ore (Pb sulphide) data, to try and define characteristic Pb isotope ranges from various lithologies of relevance to this study. We use the plotting method of Albarede et al. 2012 [21] as introduced for enamel studies [5]. This method derives the parameter of Pb model age (*T*) and ^238^U/^204^Pb (μ) and ^232^Th/^238^U (κ) ratios both of which are used to characterise metal sources.

### Geological controls on the Pb isotope compositions of rocks of Britain

**Fig 1:**
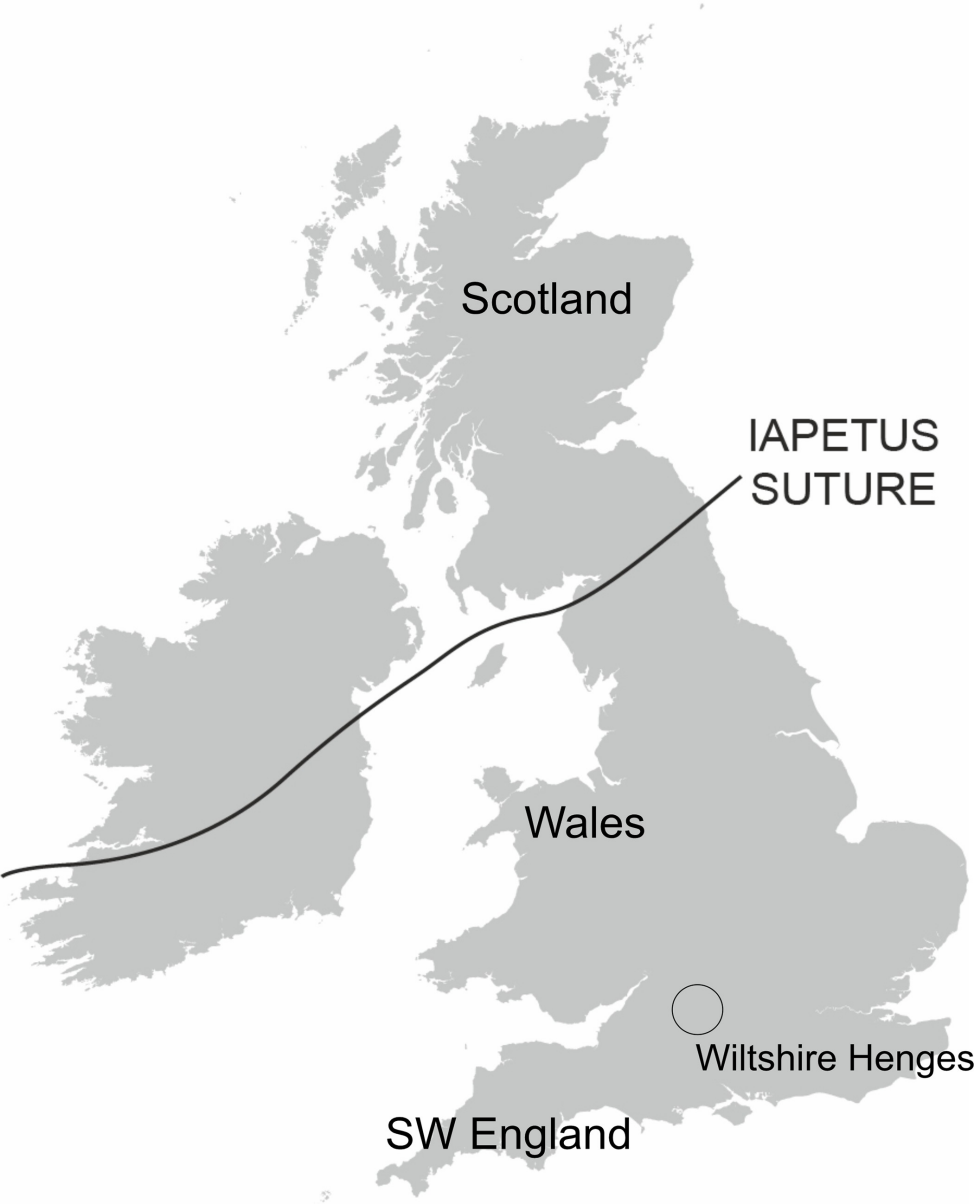
The location of the Iapetus suture.

Key to this study is our ability to distinguish between northern and southern British Pb sources and this can be achieved because of differences in the underlying geology between these two parts of Great Britain. The Pb isotope composition of rocks and minerals tends to be dominated by major geological tectonic events such as mountain building, which is accompanied by metamorphism and the intrusion of granites, the heat from which drives the re-mobilisation of Pb to create ore deposits. Thus, as described by Blicher-Toft et al. 2016 [14], Pb isotope compositions across Europe reflect large-scale tectonic events. During the Caledonian Orogeny (490- 400Ma), the tectonic plates of Laurentia and Avalonia collided as the Iapetus Ocean between them closed. The junction between these two tectonic plates (Fig 1) is called the Iapetus Suture and it runs on a NE–SW line from Berwick-upon-Tweed to the Solway Firth and projects into Ireland [22]. The underlying geology to the north and south of this suture is fundamentally different; the Laurentian basement, to the north, is geologically much older (> 3000Ma–c. 1750Ma) and is depleted in uranium (U) [23] whereas the Avalonia basement in the south is geologically much younger (c. 700Ma) [22], and this means the Pb isotope compositions, related to the basements of the two areas, are different. As this geological boundary essentially defines the modern political border of Scotland with England, it provides a potential method of discriminating between Scotland and the rest of Great Britain.

Marine carbonates, such as Chalk and limestone, often display ‘anomalous Pb’ values [10] because they are formed as precipitates from seawater, in which the U and Pb present do not retain a proportionate parent/daughter association. This is because U and Pb have very different solubility coefficients in seawater with marine carbonates accommodating an upper limit of 3 mg/kg for uranium but only 1 μg/Kg for Pb and thorium (Th). This imbalance in uptake of U and Pb can result in modern carbonates with very high ^206^Pb/^204^Pb values, which leads to a wide range of negative model ages being calculated [24]. Such features are documented in a study of Pb isotope data from Cenozoic limestones from South America [4] and in soil leach and re-equilibrated dentine values from British Chalk sites [25].

### Test case Neolithic animals

Stonehenge, and other henges in southern England were a focal point for gatherings during Neolithic times. The geographic origin of the people who created and used them is a key question to understanding their construction and use. However, Neolithic human remains are not common at many of these henges and those that are present tend to have been cremated [26], reducing the range of analytical approaches that can be employed. Consequently, studies have focused on using the feasting remains from domestic animals as a proxy for human movement. The Sr isotope values from human cremated bone are consistent with a southern British source, but bone represents a longer biological time scale and is therefore subject to biogenic averaging [27] whereas Sr isotope values suggested that Scotland could not be excluded as a source for the animals, though recent advances in mapping raise other possibilities [28]. The discovery of highly radiogenic ^87^Sr/^86^Sr values (up to 0.7172) in Neolithic animals at the Wiltshire henges has raised the issue of whether some pigs could have been brought, as livestock, from northern Britain to Wessex during the Neolithic [29, 30], a possibility that has sparked debate [31, 32]. This study explores the value of Pb isotopes to this debate.

A multi-isotope study (strontium (Sr), oxygen (O), carbon (C), nitrogen (N) and sulphur (S)) on tooth enamel from pigs observed that the animals had diverse isotope compositions (δ^34^S_VCDT_ = -1.6 to 19.6, δ^13^C_(collagen)_ = -23.1 to -19.7, δ^15^N_(collagen)_ = 3.4 to 9.1, ^87^Sr/^86^Sr: 0.7080 to 0.7172 and δ^18^O_(carbVSMOW)_ = 23.2 to 28.9). The S isotope range covers areas multiple lithologies from the coast to inland Britain and the Sr isotope values encompass virtually the full extent of the British biosphere range. There are also clear dietary differences recorded in C & N isotope data and the O isotope range (5.7‰), while not directly comparable human data, is not far from the full British human enamel range (6.4‰, n = 824) [33]. This data diversity means that there are few areas of Great Britain that can be clearly excluded for the overall source of animals found in the Neolithic henges.

Since the publication of Madgwick et al. 2019 [30] new research has uncovered new sources of highly radiogenic Sr in England. Biosphere mapping of southwest England, guided by geochemical soil profiles [34], has highlighted areas around the granite intrusions in Devon and Cornwall where Sr in plants reaches values of ^87^Sr/^86^Sr = 0.72 [28] and Roman and Medieval sheep from this region record ^87^Sr/^86^Sr ratios up to 0.717 [35, 36]. In addition, it has been shown [37, 38] that well-established forest on certain terrains, typically those with soils derived from both siliceous and carbonate bedrock, will leach out the carbonate component over time, resulting in a more radiogenic signature for the forest area. These developments mean there are now areas of England that can be considered as a possible source region as well as parts of Scotland. This paper establishes the use of Pb isotopes as a tool for discrimination between origins in northern and southern Britain.

## Material and methods

### Analytical details

The Pb analysis in this paper come from a number of sources:

1) new analyses of tooth enamel and bone from Durrington Walls, Cladh Hallan (CH) and Kings Gate, and from mineral samples from the Malvern Complex and the Lundy granite.

The tooth enamel selected was fully mineralized with good surface preservation and was prepared as follows: samples of enamel between 50-100mg were cut from the teeth using a rotary diamond wheel. The surfaces were fully abraded to a depth of 10–100 microns and all dentine was removed using a diamond burr. Bone samples were similarly abraded and cut using a rotary blade. The resulting sample was transferred to a clean (class 100, laminar flow) working area for further preparation. Samples were then cleaned by placing in >18 megaohm-cm resistivity Milli-Q water at 60°C for an hour and washed twice in Milli-Q water. They were then leached in 2% Teflon© distilled HNO_3_ for approximately 2 minutes. After a final rinse, the samples were dried and transferred into a pre-cleaned Teflon© beaker where they were dissolved in Teflon© distilled 16MHNO_3_, evaporated to dryness and converted to bromide form using Romil© UpA HBr.

The samples from the Malvern Complex and the Lundy Granite were drilled out from feldspar crystals exposed on the clean-cut surface of the hand specimens held in the National Geoscience Data Centre (NGDC). The resulting mineral powder was dissolved and dried down in Teflon distilled 29M HF and 16M HNO3 then 6MCl and then converted to Bromide form using Romil© UpA HBr.

2) We were unable to access many of the samples we wanted to analyse due to Covid departmental and museum closures. However, we found we could use residual aliquots of solutions previously prepared for Sr isotope analysis for pig [30] and sheep enamel [35, 36]. This had the advantage of minimizing unnecessary further sample damage of the teeth but meant we were restricted to those samples for which sufficient solution remained. These solutions were in chloride form and were dried down and converted to Bromide form using Romil© UpA HBr.

3) Previously published Pb analyse of pig enamel samples from Neolithic pig assemblage [5] is included in Table 1 and the preparation details for these samples are given in the source paper.

**Table 1:** Compilation of Pb and Sr isotope data from this study and previously published data. $—previously published Pb data [5], # samples solutions [30] x- samples solutions [35, 36]. Unmarked samples are new analysis from tooth enamel and bone prepared for this study.

sample	sample type	ppm	^87^Sr/^86^Sr	^206^Pb/^204^Pb	^207^Pb/^204^Pb	^208^Pb/^204^Pb	^207^Pb/^206^Pb	^208^Pb/^206^Pb	*T*	μ	κ	source
Pig enamel from Henges												
DWP04	M1	144	0.71004	18.0875	15.6150	38.0288	0.86340	2.10269	472	9.76	3.91	$
DWP05	M1	158	0.71188	18.2236	15.6237	38.1570	0.85740	2.09402	384	9.75	3.89	$
DWP07	M1	75	0.70970	18.2780	15.6247	38.1706	0.85490	2.08855	344	9.74	3.86	$
DWP10	M1	197	0.71071	18.3712	15.6498	38.2807	0.85189	2.08381	323	9.82	3.87	#
DWP13	M1	90	0.70903	18.4207	15.6338	38.3297	0.84880	2.08099	256	9.74	3.86	$
DWP15	M1	217	0.70943	18.2120	15.6295	38.1987	0.85830	2.09765	402	9.77	3.92	$
DWP20	M1	189	0.71073	18.5487	15.6566	38.3690	0.84410	2.06863	204	9.80	3.80	#
DWP22	M1	124	0.70917	18.3961	15.6312	38.3168	0.85010	2.08447	270	9.74	3.87	$
DWP24	M1	1961	0.71056	18.2378	15.6443	38.1944	0.85790	2.09469	410	9.83	3.91	$
DWP25	M1	93	0.70833	18.7106	15.6681	38.5433	0.83736	2.05995	107	9.81	3.79	#
DWP26	M1	125	0.70950	18.3790	15.6437	38.3587	0.85130	2.08753	305	9.79	3.90	$
DWP27	M1	80	0.71013	18.3942	15.6466	38.3177	0.85070	2.08359	300	9.80	3.87	$
DWP29	M1	84	0.70857	18.9283	15.6558	38.5677	0.82718	2.03801	-75	9.74	3.67	#
DWP30	M3	ND	0.70860	19.0038	15.7558	39.0624	0.82916	2.05576	61	10.10	3.90	
DWP31	M1	169	0.71721	18.4283	15.6363	38.3614	0.84849	2.08165	259	9.76	3.87	#
DWP32	M1	102	0.70937	18.2833	15.6363	38.2268	0.85530	2.09126	362	9.78	3.89	$
DWP35	M1	97	0.71032	18.3465	15.6435	38.3721	0.85280	2.09198	329	9.80	3.93	$
DWP36	M1	83	0.70956	18.2885	15.6378	38.2846	0.85510	2.09343	361	9.79	3.92	$
DWP37	M1	153	0.70975	18.2465	15.6355	38.2055	0.85690	2.09391	388	9.79	3.91	$
DWP38	M2	ND	0.71146	18.8297	15.6500	38.7124	0.83116	2.05600	-20	9.72	3.80	
DWP39	M1	99	0.71022	18.4926	15.6506	38.4216	0.84630	2.07773	234	9.79	3.86	*$*
DWP40	M1	137	0.71480	18.7752	15.6759	38.6633	0.83492	2.05931	77	9.84	3.82	#
DWP40	M2	ND	0.71919	18.5052	15.6352	38.4344	0.84491	2.07698	195	9.73	3.86	
DWP45	M1	142	0.71130	18.1385	15.6223	38.0950	0.86130	2.10028	443	9.76	3.91	$
DWP46	M1	140	0.70941	18.4686	15.6467	38.3827	0.84720	2.07833	245	9.78	3.86	$
DWP54	M1	83	0.70901	18.4676	15.6638	38.2560	0.84814	2.07154	282	9.86	3.80	#
DWP55	M1	207	0.71269	18.1633	15.6323	38.1186	0.86070	2.09873	443	9.80	3.91	$
DWP56	M1	167	0.71164	18.1463	15.6306	38.1413	0.86140	2.10194	453	9.80	3.94	$
DWP57	M1	74	0.70847	18.7911	15.6584	38.5748	0.83331	2.05290	28	9.76	3.76	#
DWP60	M1	140	0.71486	18.6615	15.6542	38.5282	0.83871	2.06475	115	9.77	3.81	#
DWP60	M2	ND	0.71376	18.6383	15.6287	38.4770	0.83852	2.06444	82	9.68	3.79	
DWP62	M1	98	0.70972	18.2598	15.6350	38.2061	0.85630	2.09243	377	9.78	3.90	$
DWP66	M1	ND	0.70816	18.8742	15.6604	38.6813	0.82973	2.04945	-32	9.75	3.76	
DWP69	M1	128	0.71025	18.5303	15.6553	38.4834	0.84500	2.07751	215	9.80	3.87	$
DWP71	M1	92	0.70917	18.3868	15.6247	38.2877	0.84980	2.08241	263	9.71	3.85	$
DWP84	M1	ND	0.70808	18.7957	15.6532	38.5906	0.83281	2.05319	12	9.74	3.76	
DWP85	M1	ND	0.70808	18.7144	15.6496	38.5562	0.83624	2.06027	67	9.74	3.79	
MD124	I	243	0.71038	18.6966	15.6455	38.4824	0.83681	2.05828	72	9.73	3.76	#
MD125	M1	133	0.71042	19.0637	15.6658	38.7316	0.82177	2.03173	-165	9.74	3.67	#
MD126	M1	ND	0.71582	18.8804	15.6560	38.6343	0.82922	2.04630	-33	9.76	3.73	
MD127	M3	13	0.70981	18.9309	15.6561	38.6943	0.82702	2.04401	-84	9.73	3.73	#
MD128	I	ND	0.71353	18.5342	15.6077	38.4433	0.84210	2.07421	120	9.62	3.83	
MD129	M1	ND	0.71404	18.8153	15.6520	38.5786	0.83187	2.05041	-4	9.73	3.74	
MD130	M1	194	0.71162	19.1770	15.6814	38.5833	0.81772	2.01199	-218	9.79	3.54	#
WK108	M1	ND	0.70822	18.9383	15.6653	38.7388	0.82718	2.04555	-59	9.78	3.75	
WK119	M1	263	0.71138	18.5046	15.6499	38.4170	0.84573	2.07611	224	9.79	3.85	#
WK120	M1	115	0.70849	18.7743	15.6564	38.5639	0.83393	2.05411	22	9.73	3.76	#
WK121	M1	215	0.71326	18.4821	15.6334	38.3186	0.84587	2.07331	209	9.73	3.81	#
WK122	M1	227	0.71014	18.0293	15.5994	37.8437	0.86523	2.09904	482	9.70	3.84	#
WK123	M1	98	0.70825	18.7726	15.6543	38.5304	0.83389	2.05251	32	9.75	3.74	#
Pig bone from Henges												
DWRM30	bone	nd	0.70768	18.8758	15.6566	38.6606	0.82945	2.04816	-41	9.74	3.75	
DWRM38	bone	nd	0.70857	18.8388	15.6540	38.6767	0.83094	2.05303	-18	9.74	3.78	
DWRM60	bone	nd	0.70950	18.7552	15.6398	38.5810	0.83390	2.05711	16	9.70	3.78	
SW England Roman/Medieval sheep												
EX-Med1-Ovis4				18.4321	15.6327	38.4054	0.84810	2.08365	245	9.73	3.89	x
Ex-Er-Ovis2				18.4351	15.6361	38.4203	0.84820	2.08411	249	9.75	3.90	x
Ex-Med3-Ovis9				18.4766	15.6405	38.4574	0.84650	2.08144	227	9.75	3.89	x
EX-RLF-Ovis6				18.4369	15.6476	38.4738	0.84870	2.08681	270	9.79	3.93	x
Ex-Med3-Ovis7				18.4991	15.6452	38.4843	0.84570	2.08036	219	9.77	3.89	x
EX-Med1-Ovis6				18.3998	15.6374	38.4211	0.84990	2.08815	278	9.76	3.92	x
Ex-Med4-Ovis4				18.4311	15.6243	38.4360	0.84770	2.08542	230	9.70	3.90	x
Ex-Med2-Ovis7				18.5386	15.6422	38.4992	0.84380	2.07674	184	9.75	3.87	x
EX-Med1-Ovis5				18.4398	15.6379	38.4172	0.84810	2.08342	249	9.75	3.89	x
Ex-Med2-Ovis12				18.4866	15.6421	38.4696	0.84610	2.08097	223	9.76	3.89	x
Ex-Er-Ovis11				18.4857	15.6825	38.5954	0.84836	2.08807	294	9.91	3.97	x
Ex-Med2-Ovis11				18.4106	15.6333	38.4318	0.84910	2.08751	262	9.74	3.92	x
Ex-LR-Ovis1				18.4496	15.6394	38.4381	0.84770	2.08344	245	9.76	3.90	x
Ex-Med4-Ovis3				18.4936	15.6340	38.4824	0.84540	2.08088	201	9.73	3.89	x
Ex-Med2-Ovis4				18.4629	15.6383	38.4475	0.84700	2.08244	233	9.75	3.89	x
Ex-Med2-Ovis2				18.5305	15.6510	38.5369	0.84461	2.07975	208	9.79	3.90	x
Ex-Med4-Ovis2				18.4915	15.6409	38.4785	0.84580	2.08090	220	9.76	3.89	x
Ex-Er-ovis06				18.3385	15.6251	38.3340	0.85200	2.09038	300	9.72	3.91	x
Ex-Er-ovis03				18.4051	15.6274	38.4042	0.84910	2.08664	255	9.72	3.90	x
Ex-Er-ovis04				18.4499	15.6402	38.4655	0.84770	2.08490	246	9.76	3.91	x
Malvern Precambrian Igneous Complex												
MR 35599	feldspar			18.1005	15.6162	37.8133	0.86275	2.08908	460	9.75	3.78	
MR 8625	feldspar			18.1043	15.6267	37.7799	0.86315	2.08679	477	9.79	3.76	
Lundy Tertiary Granite												
MR31924 01	feldspar			18.9863	15.6572	39.0150	0.82466	2.05490	-124	9.72	3.85	
MR31924 02	feldspar			19.0036	15.6597	39.1050	0.82404	2.05777	-132	9.73	3.89	
MR31924 03	feldspar			18.8821	15.6511	38.9350	0.82889	2.06201	-57	9.72	3.88	
MR31924 04	feldspar			18.7627	15.6408	38.8100	0.83361	2.06847	12	9.70	3.89	
MR31924 05	feldspar			18.8803	15.6500	38.9280	0.82891	2.06183	-58	9.71	3.87	
MR31924 06	feldspar			18.9569	15.6586	39.10654	0.82601	2.06292	-98	9.73	3.92	
MR31924 07	feldspar			18.9357	15.6586	39.08357	0.82694	2.06402	-82	9.74	3.92	
MR31924 08	feldspar			18.9663	15.6600	39.11559	0.82568	2.06237	-103	9.74	3.91	
MR31924 09	feldspar			18.9508	15.6591	39.09103	0.82630	2.06276	-93	9.74	3.91	
MR31924 10	feldspar			19.0258	15.6613	39.11122	0.82316	2.05569	-145	9.73	3.88	
MR31924 11	feldspar			18.9477	15.6596	39.133	0.82646	2.06532	-89	9.74	3.93	
MR31924 12	feldspar			18.9757	15.6603	39.14144	0.82528	2.06272	-109	9.74	3.92	
Scotland, Bronze Age sheep/goats												
CH 412 (M1/2)				18.456	15.558	39.178	0.84298	2.12281	78	9.44	4.25	
CH 412 (M1/2)				18.471	15.578	39.046	0.84338	2.11396	108	9.51	4.18	
CH 595 (M1)				18.574	15.587	38.969	0.83917	2.09808	46	9.53	4.07	
England, Neolithic human												
63262		149	0.70887	18.8026	15.6409	38.6542	0.8318	2.05570	-2.535	9.72	3.79	

Separation of Pb from samples was undertaken using standard AG-1 X8 ion exchange techniques. In some cases, the residual solution from the AG-1 X8 column was converted to chloride and passed through an Eichrom© 50AG X8 column to separate out Sr.

Contribution of laboratory blank to analysis. The laboratory Pb blank is between 5-30pg with a fall in blank composition, measured on the clean lab windowsill outwith the laminar flow hoods, of ^206^Pb/^204^Pb = 17.72. Therefore a 50-milligram sample with 0.1ppm Pb, combined with 30pg blank contribution would provide a worst-case blank contribution of 0.6%. For a sample with a ^206^Pb/^204^Pb ratio of 18.80 this would result in a shift of -0.01 and is therefore negligible for the purposes of this study.

Samples analysed by Thermo Fisher Scientific Neptune Plus MC-ICP-MS were spiked with a thallium (Tl) solution and introduced into the instrument via an ESI 50 μl/min PFA micro-concentric nebuliser attached to a de-solvating unit (Cetac Aridus II) and normalised to NBS981 [39]. Average 2 SD reproducibility for the following ratios was ^206^Pb/^204^Pb  =  0.008%; ^207^Pb/^204^Pb  =  0.008%; ^208^Pb/^204^Pb  =  0.009%.

Sr was loaded onto a single Re Filament [40] and the isotope composition and Sr concentrations were determined by Thermal Ionisation Mass Spectrometry (TIMS) using a Thermo Triton multi-collector mass spectrometer. The international standard for ^87^Sr/^86^Sr, NBS987, gave a value of 0.710262 ± .000020 (2SD, n = 8) during the analysis of these samples. Data are corrected to an accepted value of for NBS987 of 0.710250.

## Results

### A new Pb iso-scape for Great Britain

**Fig 2:**
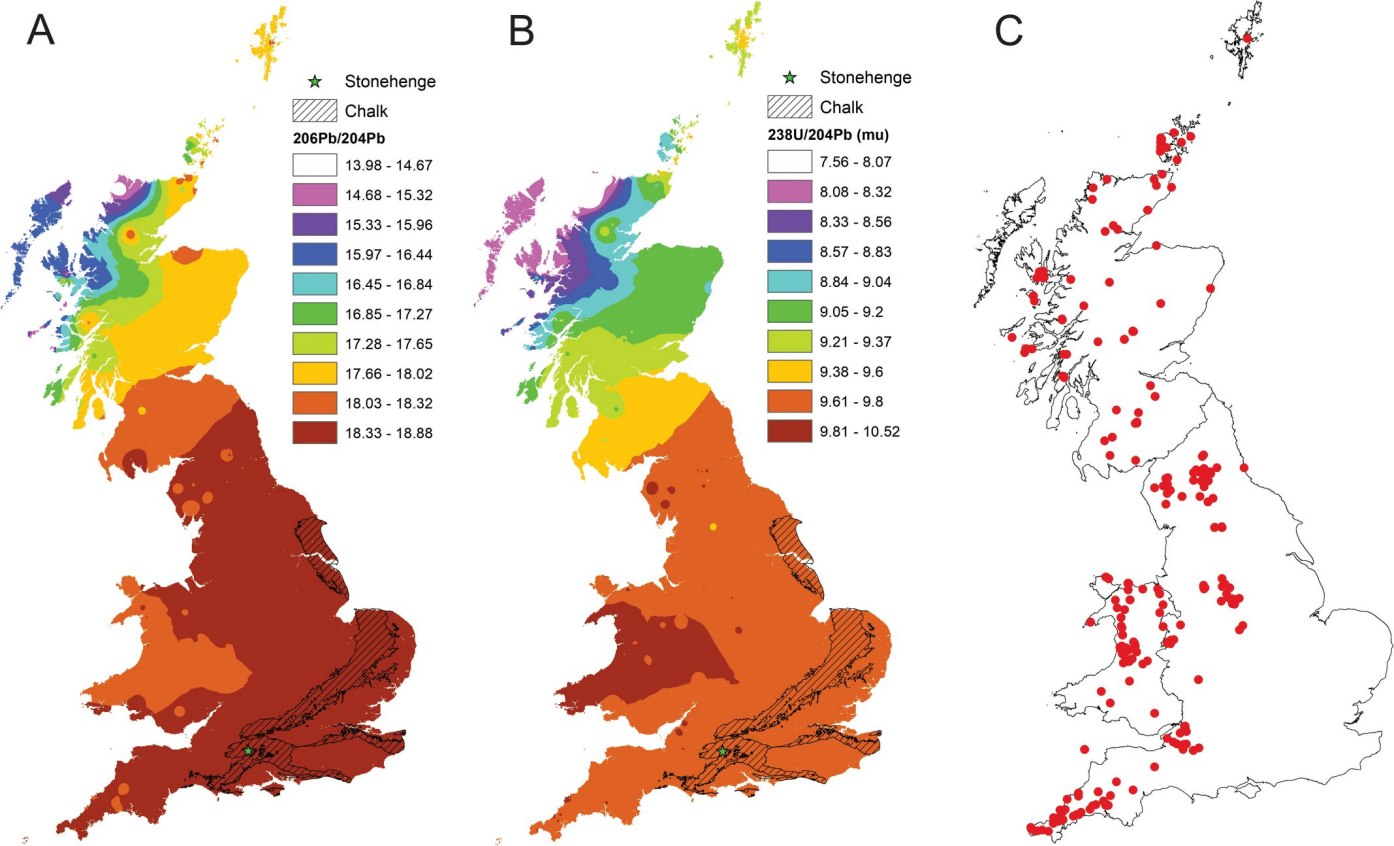
A contoured map of A) ^206^Pb/^204^Pb isotope compositions B) ^238^U/^204^Pb (μ) values from Great Britain C) samples locations. Data for this plot are taken from the compilation of [14–16, 19, 41] and this study. Contouring is based on Inverse Distance Weighting (IDW) with ten natural break intervals. Superimposed over this contour map is the outcrop area of Chalk. This has been designated data ranges of ^206^Pb/^204^Pb 18.81 ±0.2(1SD, n = 26) and ^238^U/^204^Pb (μ) = 9.71±0.24(1SD, n = 26). Contains OS data © Crown copyright and database rights 2022.

A Pb isotope zonation map of Britain has been created which highlights the tectonically controlled geospatial variation in Pb isotope values across Britain using published data [14–18, 20] and data from this study. It is contoured using inverse distance weighting (IDW) with ten natural break categories for ^206^Pb/^204^Pb; and ^238^U/^204^Pb (μ) which are shown in Fig 2 alongside the sample sites (n = 633). The maps show the strong zonation of the isotope compositions across the Iapetus Suture and throughout Scotland which reflects the influence of the very old Laurentian basement that underlies much of Scotland. England is dominated by Hercynian mineralisation focussed on the Pennines, Mendips, and SW England ore fields whereas the ores in Wales have an older Avalonian signature.

Superimposed over this map is an outline of Chalk outcrop. This lithology is an important substrate in southern England and its domain is designated an isotope range of ^206^Pb/^204^Pb 18.815 ±0.0195(n = 26) and ^238^U/^204^Pb (μ) of 9.71±0.24 (1SD, n = 26), using data from this study and Montgomery (2002) [25].

### The construction of reference data fields

We have created four reference data fields in μ- *T* space, representing the main geographic Pb isotope sub-divisions defined in Fig 2. The co-ordinates for the field can be obtained from J. Evans.

Ores from Scotland: The data from Scotland define a broad sloping field from c 70Ma—2400 Ma [14]. We focus on the younger end of this array which is constructed using the following data sources [14–17, 19, 41]. The boundary lines for this field encompasses 90% of these data.

Ores from Wales: The 1SD field for *T* vs μ, for data from Wales is taken from published studies [14, 41] and includes samples of Avonian basement from the Malverns Complex (Table 1).

Ores from England: The Pennines, Mendips and south-west England mineralization are all similar geological age. There is a large amount of data published from these areas [14, 20, 42]. The data field is based on the 1SD range of data (n = 197).

The Chalk: This is the rock that underlies much of southern Britain and is an important lithology for archaeological studies because it is both a focus of important Neolithic and Bronze Age sites and because of its preservation properties. The Chalk does not host economic Pb mineralisation so there is an absence of published Pb isotope data in the geological literature. We have derived the 1SD range for T and μ from Pb isotope analysis of Chalk soil leaches and equilibrated dentine [25], and bone samples (this study).

### Testing the validity of using mineral composition as a proxy for bioavailable Pb

**Fig 3:**
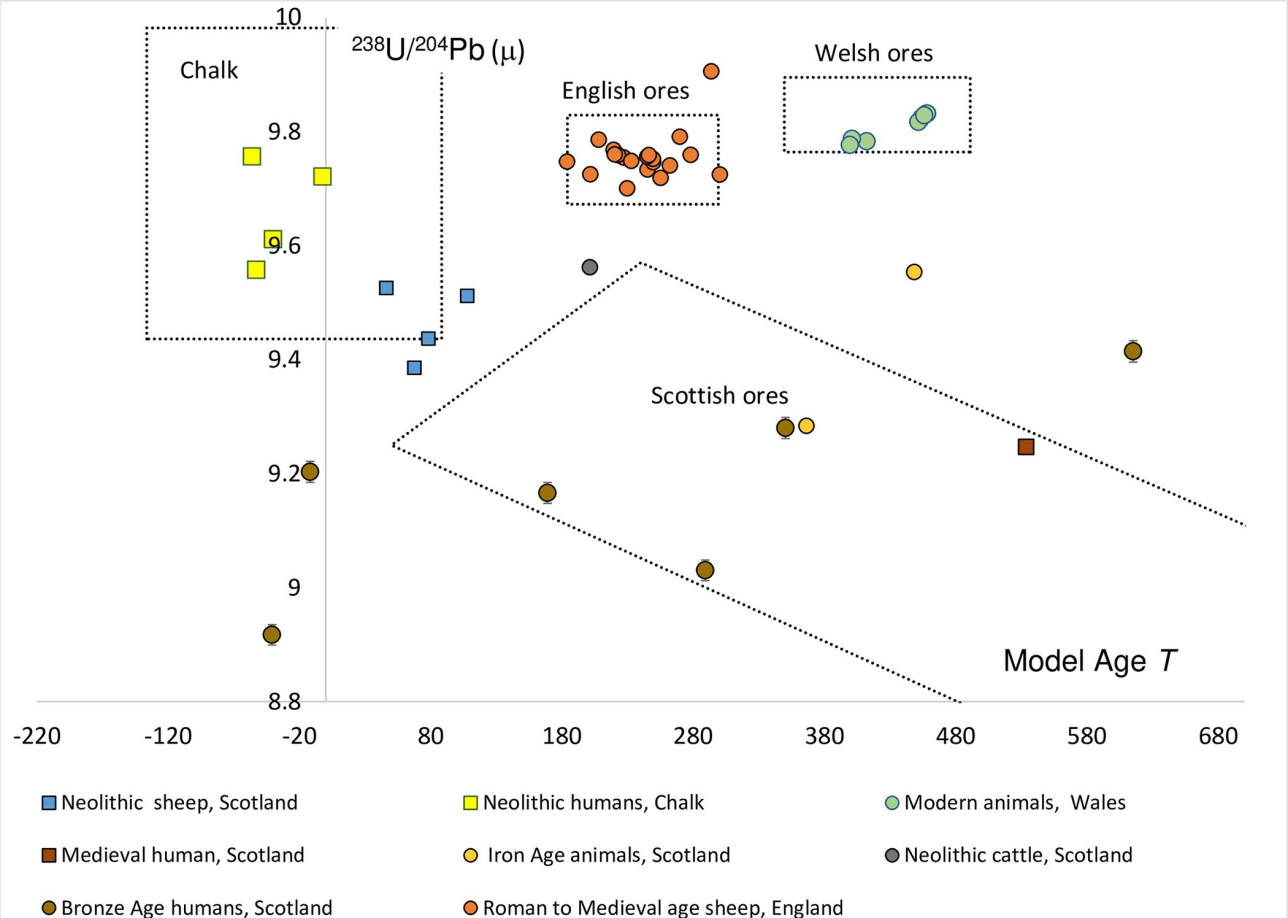
Validation of mineral data fields using published human and faunal sample data sources [11, 25, 43, 44] and data from this study.

The transmission of these geological signals into the biosphere is critical for the application of this method to provenance studies. Fig 3 shows the reference fields in *T* vs μ space with archaeological faunal/human data to validate them. This exercise is limited by the paucity of biosphere data currently available. However, there is sufficient data to demonstrate the assumption that, in a situation of geogenic exposure, the faunal/human data reflect the labile Pb isotope composition of the local geological environment.

There are two sites where Pb isotope analyses are available from pre-mining (Neolithic) human enamel [45, 46]. The Sr isotope composition of the tooth enamel has been used to select those individuals that had a childhood origin on Chalk. These include three juveniles [45] and one adult [46]. These four samples have Pb isotope compositions that plot within the 1SD field designated to represent the Pb isotope composition of the Chalk and thus supports the transfer of the geogenic Pb composition into the tooth enamel.

The validation of the ore field in England is based on new data presented in this study from south-west England. The samples are from sheep/goat samples of Roman [36] and Medieval [35] age excavated in Exeter [47]. The Pb data from these animals plot as a cluster within 1SD of the Pb isotope field, thus showing the relationship between the Pb isotope compositions of the mineralization in England and the faunal biosphere.

Measurements of modern animals provide evidence for transmission of geogenic Pb isotope composition from ore field for Wales into the biosphere field. These samples were specifically collected from Wales in areas where Pb mining is extensive. Consequently, natural Pb levels are high and the influence of modern pollution is swamped by the geogenic component in the environment [43]. The data plot well within the 1SD data field based on mineral data from Wales.

The data used to validate the transmission Pb isotope compositions between the geogenic source and over lying biosphere for the ore field for Scotland comes from sites on the Hebrides and Orkney and both human and animal samples have been used to try and characterize this data field. All the samples have low Pb concentrations consistent with only geogenic lead exposure and/or predate anthropogenic lead release. The data scatter and human samples show considerable variation in μ and Pb model ages. Unlike the previous three data guidance fields for Chalk, England and Wales ores, the Pb isotope composition of the biosphere samples does not plot neatly within the ore field for Scotland, however, these samples show two distinguishing features. All samples have low μ values, between 9.6 and 8.9, and they also have elevated κ values >4.0 which are typical of ores from Scotland [14]. It is hoped that future studies can develop this validation of the dataset from Scotland with well provenanced samples, free from modern anthropogenic contamination, however, preservation is a problem in large parts of Scotland. We recommend that, while this method can be used to distinguish between sources in northern and southern Britain, the transmission of the high resolution Pb isotope zones seen in the mineral data (Fig 3) cannot currently be demonstrated in biosphere samples.

In summary, reference data fields for the Pb isotope composition of geographical areas of Britain, have been defined with geological data and the transfer of these signatures into the biosphere has been validated with well-provenanced faunal data. It should be noted that as most of the fields are defined at 1SD, which represent c. 66% of the reference data, plotting outside the field only excludes samples at this level of certainty. With this validation in place, the data from the pigs can be examined.

## Discussion

### Test case using pig enamel samples

**Fig 4:**
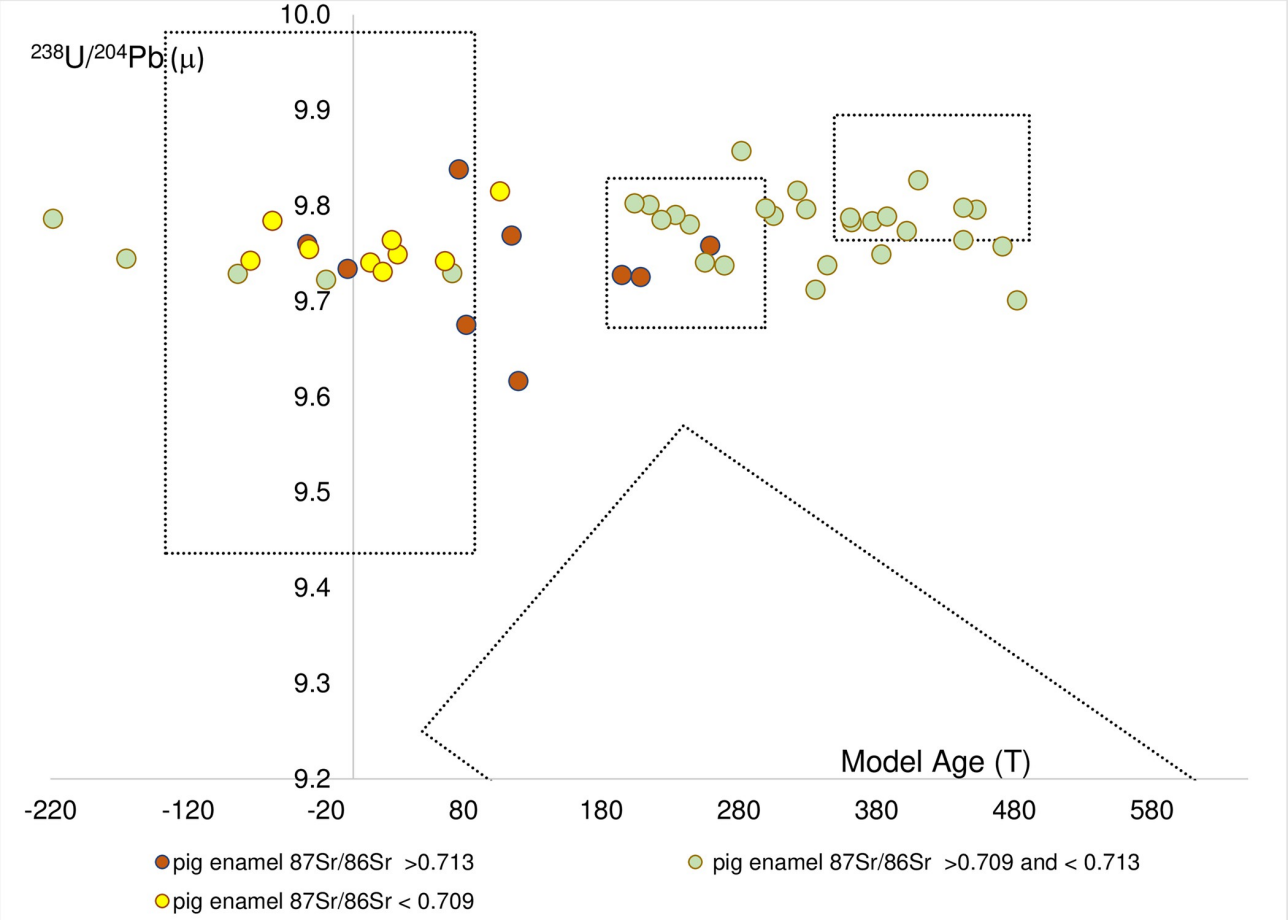
The pig tooth enamel from the henges shown in relationship to the mineral reference fields. The data are colour-coded based on strontium isotope ranges of the enamel.

The Pb isotope compositions of the pig enamel are presented in Table 1 and plotted in the form of μ versus *T* in Fig 4. The data points are colour-coded to represent ^87^Sr/^86^Sr isotope composition groupings of the data: <0.709, 0.709–0.713, and > 0.713. These Sr groups broadly represent a) carbonates terrains, b) most silicate terrains and c) Rb-rich/very old terrains, respectively.

The Pb isotope composition from the pig enamel samples, have model ages that range between the negative values of the southern Britain Chalk field to the Palaeozoic model ages as old as 482 Ma, and all have μ values above 9.62. The μ values excludes them from the field of ore data from Scotland and hence the Pb isotope data indicate that these animals which represent a 36% subset of those in Madgwick et al. 2019 [30], are inconsistent with an origin in Scotland.

Around half the samples have model ages that plot between 195 Ma and 482 Ma, indicating Pb sources from a Palaeozoic/Late Precambrian rocks substrates and three of the samples (DW31, DW40 and DW60) with radiogenic values (> 0.713) plot in the data field for Southwest and central England and hence could be sourced from these areas. Nine of the samples with ^87^Sr/^86^Sr < 0.709 have both Pb and Sr isotope characteristics consistent with an upbringing on a Chalk terrain (Fig 4). Five samples (four from Marden [MD 124, 125, 127, and 130] and one from Durrington Walls [DW 38]) have Sr values between 0.7098–0.7116 and plot in a sub-linear array across the southern Britain Chalk field. This combination of carbonate dominated Pb values, but Sr isotope values above those generally attributable to pure Chalk, could suggest that the animals were raised on a less pure carbonate lithology such as the Jurassic Limestone or a mixed silicate -carbonate sediment.

Having established that many of the samples record combined Sr and Pb isotope compositions that can be accommodated by known tectonic and lithological domains found in Britain, we are left with a few samples for which the data are more complex. These samples record elevated radiogenic composition for both Sr and Pb. They are referred to as the “anomalous group” and are discussed below.

### The relationship between the Sr and Pb isotope compositions in tooth enamel

Although both biosphere Sr and Pb are ultimately sourced from a geological origin it should not be expected that there will be a direct correlation between the two systems; they will reflect different aspects of the source biosphere. A carbonate terrain has restricted and relatively low Sr isotope compositions, due to the very low rubidium (Rb) content of limestone, whereas the Pb isotopes may range in values considerably but will tend to have elevated ^204^Pb/^206^Pb values as a consequence of high and variable time-integrated U/Pb in the limestones [4]. This will lead to high μ values and negative model ages [21].

In silicate terrains the relationships between Sr and Pb isotopes are the inverse of carbonates terrains. The variable lithologies have a wide range of Rb/Sr ratios leading to transmission of wide-ranging ^87^Sr/^86^Sr values into the biosphere whereas the Pb isotopes will reflect the overall tectonic environment.

There are six samples (three animals from Marden and two animals (3 teeth) from Durrington Walls [MD126, MD 128, MD129, DW40-M1, DW60- M1&M3] with both high Sr (^87^Sr/^86^Sr > 0.713) and Pb values (^206^Pb/^204^Pb > 18.7) which generate μ values of >9.6 and young Pb model ages between 30 Ma and 123 Ma (Fig 4). This combination of radiogenic Pb and Sr isotope features is very difficult to reconcile with a single lithological source and essentially suggests a Tertiary source with highly radiogenic Sr composition. Such a rock type does occur in the form of the small island of Lundy (4.5km^2^) off the north Devon coast [48]. While we cannot exclude this granite zone as a possible origin, and it is known to have been inhabited by Neolithic communities [49], it would not be parsimonious to assign origins for the anomalous pig samples to Lundy, based on the island’s small size, and relative remoteness

Several possible alternative explanations can be considered. The isotope signal could derive from an upbringing on a sedimentary rock composed of old/Rb rich silicate minerals in a carbonate matrix where the Sr was predominantly derived from the silicate component whereas the Pb was predominantly from the carbonate matrix. However, this isotope mismatch could also reflect metabolic discrimination rather than a simple foddering source. It is known that Pb can be reactivated into the blood stream during times of stress [50], so it is possible that this isotope signal could be caused by mobility, foddering or stress that disconnects the sources of Pb and Sr from different body reserves during the formation of enamel in the animals. Understanding these possible processes is beyond the remit of this paper and will involve detailed investigation on the nature and relationship of Pb and Sr uptake during enamel formation.

## Conclusions

It has been frequently stated, and needs to be re-iterated, that isotope methods can only exclude possible areas of origin and, added to that, can only exclude options for which reference data are available, reliable and appropriate.

This paper develops the use of Pb isotopes as a further isotope discriminant for biosphere samples that have not been affected by anthropogenic pollution; either pre-metallurgical populations or those with enamel samples where Pb concentrations below the threshold for natural exposure in skeletal studies of 0.5 [11] or 0.7 mg/kg [51] in [human] enamel.

We have produced the first Pb isoscape map for Great Britain which provides a geographic distribution of Pb mineralization events and also characterizes the Chalk, which is an important lithology for archaeological studies. These main subdivisions are defined in *T*-μ space and have been validated for biosphere use with human and faunal data.

This study highlights the way in which Pb isotope domains are associated with large tectonic terranes and, because to the tectonic make-up of Great Britain, provides a powerful tool for discrimination between biosphere in Scotland (the Laurentian basement) and southern Britain (the Avalonia basement).

We have tested this application using a sample of Neolithic pig enamel from sites in southern England, some of which, because of Sr isotope composition, could not be excluded from an origin in northern Britain. Pb isotope data from the teeth excludes Scotland as a source but the diverse range of Pb isotope results, combined with other isotope proxies, are consistent with the animals being raised on a variety of lithologies of diverse age and from variable environments.

The study also highlights the fact that the uptake of both Sr and Pb may not always be consanguineous as some samples display a combination of Sr and Pb isotope signatures that are currently difficult to reconcile with a single geographic/geological origin.

This study demonstrates the diagnostic potential of Pb isotope compositions as a provenance tool in samples unaffected by anthropogenic Pb exposure and highlights the different scale of discrimination between tectonic terrains which Pb isotopes provide in comparison to the lithological scale of Sr isotope data.

## Supporting information

S1 Data (XLSX)
